# Characterization of Fatty Acid Composition Underlying Hypothalamic Inflammation in Aged Mice

**DOI:** 10.3390/molecules25143170

**Published:** 2020-07-11

**Authors:** Ye Jin Kim, Thai Hien Tu, Sunggu Yang, Jae Kwang Kim, Jae Geun Kim

**Affiliations:** 1 Division of Life Sciences, College of Life Sciences and Bioengineering, Incheon National University, Incheon 406–772, Korea; 201721047@inu.ac.kr (Y.J.K.); thaihientu@gmail.com (T.H.T.); 2 Department of Nano-Bioengineering, Incheon National University, Incheon 406–772, Korea; abiyang9@gmail.com; 3 Division of Life Sciences and Bio-Resource and Environmental Center, Incheon National University, Incheon 406–772, Korea

**Keywords:** hypothalamus, inflammation, aging process, fatty acids, fatty acid utilization

## Abstract

Degenerative diseases, which can develop during aging, are underlined by inflammatory processes. Hypothalamic inflammation triggered by elevation in circulating fatty acid levels is directly coupled to metabolic disorders. The present study aimed to investigate and characterize the hypothalamic inflammation and composition of fatty acids in the hypothalami of aged mice. We verified that inflammation and microglial activation occur in the hypothalami of aged mice by performing quantitative real-time PCR and using immunohistochemistry methods. In addition, we observed increased levels of various saturated fatty acids in the hypothalami of aged mice, whereas no major changes in the levels of circulating fatty acids were detected using gas chromatography with a flame ionization detector. Collectively, our current findings suggest that increases in saturated fatty acid levels are coupled to hypothalamic inflammation and thereby cause perturbations in energy metabolism during the aging process.

## 1. Introduction

During aging, multiple cellular stresses such as inflammation, oxidative stress, and endoplasmic reticulum stress affect the central nervous system (CNS) [1,2,3,4]. In particular, elevated inflammatory responses in the brain are common during aging [1,5]. Multiple lines of evidence have also suggested that brain inflammation accompanied by reactive gliosis is directly coupled to the development of neurodegenerative diseases such as Alzheimer’s disease, Parkinson’s disease, dementia, and amyotrophic lateral sclerosis [6,7]. Thus, it is currently accepted that mitigation of brain inflammation could be an effective strategy for treatment of neurodegenerative diseases. Inflammation is an important contributor to the development of age-associated metabolic disorders such as obesity, diabetes, and atherosclerosis [8,9]. Hypothalamic inflammation is one of the critical pathogenic components in the deterioration of hypothalamic circuit activity, causing development of obesity and its secondary complications [8,10,11,12]. 

Increases in levels of adipokines and nutrients have been linked to adiposity condition, characterized by elevated hypothalamic inflammation and disturbed energy homeostasis. Furthermore, increased levels of circulating saturated fatty acids (sFAs) during over-nutrition participate in the initiation of hypothalamic inflammation [13]. A growing body of evidence has suggested that hypothalamic reactive gliosis accompanied by inflammation is a pivotal cellular event during the development of obesity in association with over-nutrition [9,11]. In addition, we recently reported that short-term exposure to a high-fat diet (HFD) leads to increases in sFA levels in the hypothalamus [14]. However, the hypothalamic FA profile during aging remains poorly studied. 

Our present study aimed to investigate the alterations in FAs levels in the hypothalami of aged mice and to determine the possible impact on the hypothalamic inflammation linked to metabolic abnormalities during aging. In line with the notion that activated microglia dynamically participate in the initiation of hypothalamic inflammation, which is involved in obesity pathogenesis [12,15], we first confirmed that inflammation occurs in the hypothalamic microglial cells of aged mice. Furthermore, we monitored the levels of FAs in both hypothalamic tissues and sera of the aged mice.

## 2. Results

### 2.1. Aged Mice Are Characterized by Enhanced Hypothalamic Inflammation

**Figure 1:**
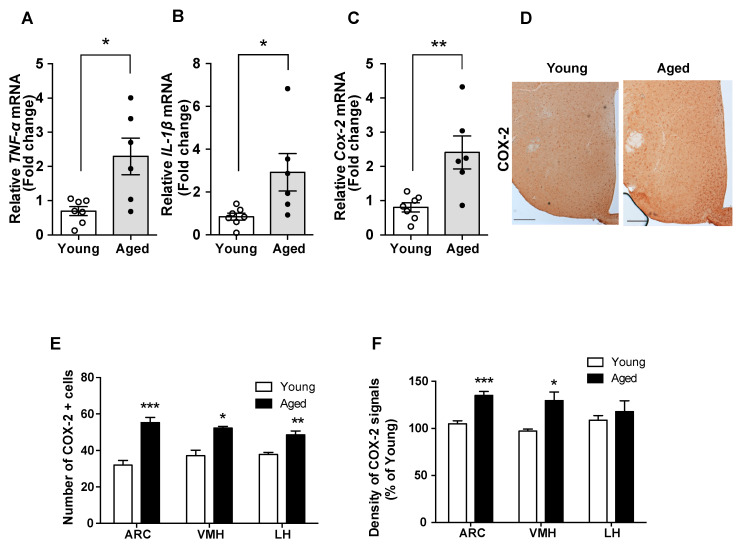
Enhanced hypothalamic inflammation occurred in the aged mice. The comparison between young and aged C57BL/6 mice revealed that the mRNA levels of genes involved in inflammation, such as (**A**) *TNF-α*, (**B**) *IL-1β*, and (**C**) *COX-2*, are increased in hypothalamic samples from aged mice. (**D**) Representative images showing the immunosignals of COX-2 in the hypothalamus of young and aged mice. Quantification of (**E**) the number of COX-2-positive cells and (**F**) the intensity of COX-2 immunoreactive signals in hypothalamic nuclei such as the arcuate nucleus (ARC), ventromedial nucleus of the hypothalamus (VMH), and lateral hypothalamus (LH) reveals that these parameters are higher in aged mice compared with young mice. The results are presented as the means ± SEMs. *n* = 6–7 for each group. * *p* < 0.05, ** *p* < 0.01, *** *p* < 0.001 for the aged group versus the young group. Scale bar = 100 μm.

Hypothalamic inflammation is regarded as a primary cause of obesity pathogenesis [9,16]. Here, we compared inflammatory responses in the hypothalamus of young and aged mice. In aged mice, we found increased mRNA levels of pro-inflammatory cytokines (such as *TNF-α* and *IL-1β*) and *cyclooxygenase-2 (COX-2)* mRNA, a key enzyme in the production of prostaglandins, which also participate in cellular inflammatory responses (Figure 1A–C). In addition, the aged mice displayed elevated number of COX-2-positive cells and higher COX-2 immunoreactivity in the hypothalamic nuclei such as the arcuate nucleus (ARC), ventromedial nucleus of the hypothalamus (VMH), and lateral hypothalamus (LH) (Figure 1D–F). Collectively, these data suggest hypothalamic inflammation in aged mice.

### 2.2. Hypothalamic Microgliosis Occurs in Aged Mice

**Figure 2:**
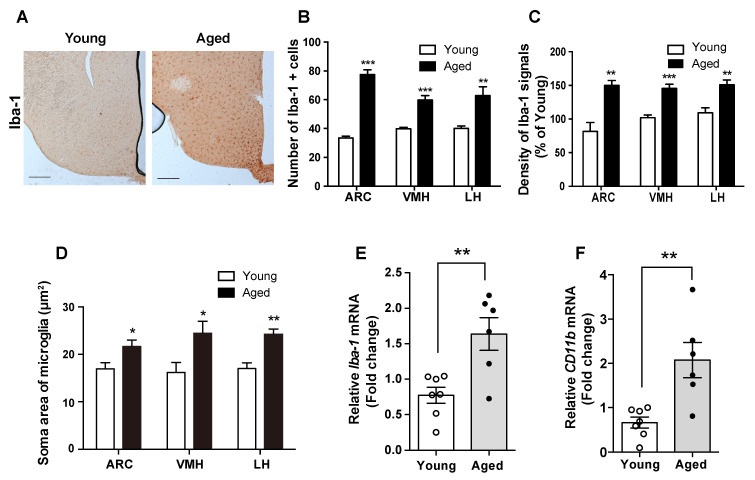
Aged mice display microglial activation and inflammation in the hypothalamus. Hypothalamic sections from young and aged C57BL/6 mice were subjected to IHC. (**A**) Representative images showing the immunosignals of Iba-1 in the hypothalamus of young and old mice. Increases in (**B**) the number of microglial cells, (**C**) the intensity of Iba-1 immunoreactive signals, and (**D**) the soma area of microglial cells were observed in the ARC, VMH, and LH of the aged mice compared with young mice. The mRNA levels of (**E**) *Iba-1* and (**F**) *CD11b* were significantly increased in the hypothalamus of the aged mice compared with those in the young mice. The results are presented as the means ± SEMs. *n* = 6–7 for each group. * *p* < 0.05, ** *p* < 0.01, *** *p* < 0.001 for the aged group versus the young group. Scale bar = 100 μm.

Different studies have suggested that the microglia act as dynamical modulators of CNS inflammation [12,15]. To test this model, we studied the level of the Iba-1 protein, which is a molecular marker for active microglia, where it participates in membrane ruffling and phagocytosis. Compared with young mice, in aged mice, we observed increased level of the Iba-1 protein in the microglia, determined by counting Iba-1-positive cells and detecting a higher intensity of Iba-1 immunosignals in multiple hypothalamic nuclei including ARC, VMH, and LH (Figure 2A–C). In addition, the aged mice displayed expanded soma areas of microglia in the hypothalamic nuclei such as ARC, VMH, and LH (Figure 2D). These findings were confirmed by the observed elevation in the mRNA levels of *Iba-1* and *CD11b* in the hypothalamus of aged mice compared with young mice (Figure 2E,F). These findings indicate that the hypothalamic inflammation during aging occurs in the microglial cells.

### 2.3. Aged Mice Display Elevation in Hypothalamic sFA Levels

Previous reports have shown that alterations in FA composition are tightly coupled to the pathogenesis of human diseases occurring during aging [17]. It has been well established that changes in circulating FA levels have an impact on the hypothalamic function and are involved in the development of metabolic disorders [18]. Thus, we monitored the levels of FA in the hypothalamus, which is a center for the control of energy homeostasis. Compared with young mice, the aged mice displayed higher levels of sFAs in the hypothalamus, including myristic acid, palmitic acid, linoleic acid, γ-linolenic acid, and arachidic acid. Furthermore, the levels of unsaturated FAs (uFAs) such as oleic acid were lower in aged mice compared with young mice (Table 1). These observations are consistent with the notions that sFAs serve as triggers of inflammation, while uFAs serve as triggers of anti-inflammatory responses. Intriguingly, linoleic acid, which is an uFA, was significantly increased in hypothalami of the aged mice when compared with young mice (Table 1). This unexpected result might be a homeostatic cellular response to mitigate the inflammation triggered by sFAs. In order to further confirm the interrelationship between levels of hypothalamic and circulating FAs during the aging, we tested the levels of FAs in serum from both young and aged mice. No significant difference between the serum FAs levels in young and aged mice was observed (Table 1). These findings suggest that altered composition of hypothalamic FAs is associated with the aging-related hypothalamic inflammation.

Aged mice show alterations in the long chain fatty acid profile in the hypothalamus. Fatty acid profiling of the hypothalamus of young and aged C57BL/6 mice was performed using gas chromatography with flame ionization detection (GC-FID). Elevation of C14:0, C16:0, C18:2n6, C18:3n6, and C20:0 levels in the total hypothalami of aged mice compared with those in young mice was observed. Aged mice displayed reduction in the C18:1n9 level in the total hypothalami compared with those in young mice. Plasma levels of fatty acids were evaluated in both young and aged mice using GC-FID. Results are presented as the mean ± SEM. * p < 0.05, ** p < 0.01 and *** p < 0.001 vs. young mice as a control.

### 2.4. Enhanced Fatty Acid Utilization Is Observed in the Hypothalami of Aged Mice

**Figure 3:**
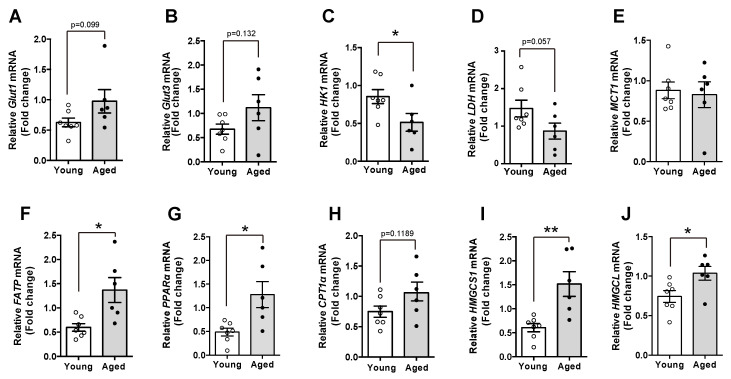
Aged mice display alternations in the expression of genes involved in cellular metabolism. mRNA expression in the hypothalamus of both young and aged C57BL/6 mice was analyzed. The mRNA levels of (**A**) *glucose transporter-1 (Glut1)* and (**B**) *Glut3* were elevated in the hypothalamus of aged mice compared with young mice. Aged mice showed decreased levels of hypothalamic genes including (**C**) *HK1* and (**D**) *LDH*. (**E**) No significant differences in hypothalamic *MCT1* mRNA were observed between young and aged mice. The mRNA levels of (**F**) *FATP*, (**G**) *PPAR-α*, (**H**) *CPT1α*, (**I**) *HMGCS1*, and (**J**) *HMGCL* genes were increased in the hypothalami of the aged mice compared with young mice. Results are presented as mean ± SEM. *n* = 6–7 for each group. * *p* < 0.05, ** *p* < 0.01 for the aged group versus the young group.

To explore the molecular mechanism accounting for the changes in FAs during the aging, we evaluated mRNA levels of multiple genes involved in cellular metabolism. We first observed that in contrast to young mice, aged mice displayed higher levels of *glucose transporter 1* and *3*, which are the functional types in glial cells (Figure 3A,B). In addition, we confirmed reduced glycolytic activity and lactate synthesis through identifying reduced mRNA levels of *HK1*, an enzyme triggering glycolysis and *LDH*, an enzyme for synthesis of lactate. These results suggest that glucose utilization was declined in the hypothalami of aged mice compared with young mice (Figure 3C,D). However, we could not show a significant difference in the *MCT-1* mRNA level, a transporter of monocarboxylate, in the aged hypothalamus (Figure 3E). Intriguingly, aged mice had higher mRNA levels of genes involved in FAs utilization such as *FATP*, a fatty acid transporter and *PPAR-α* and *CPT1-α*, the major regulators of long chain fatty acids (LCFAs) oxidation, than young mice, indicating that the aged hypothalamus retains enhanced FAs utilization (Figure 3F–H). Moreover, we also found that aged mice showed increased mRNA levels of genes involved in ketogenesis such as *HMGCS1* and *HMGCL* compared with young mice (Figure 3I,J). These results suggest that increased levels of FAs are associated with enhanced FAs utilization, which might be an alternative response to the limited glucose utilization in the hypothalamus.

## 3. Discussion

The main findings of this study are that hypothalamic inflammation increases during aging and that this is accompanied by alterations in the LCFAs profile in the hypothalamus. In addition to revealing that the enhanced aged-related inflammation occurs in hypothalamic microglial cells, we reported increased levels of LCFAs in the aged hypothalamus. This finding could be crucial for better future understanding of the underlying mechanism of intracellular metabolic processes linked to disrupted hypothalamic control of whole-body metabolism during aging. A growing body of evidence has suggested that multiple degenerative diseases correlating with senescence were directly associated with cellular inflammation [19]. Furthermore, the proportion of the patients retaining metabolic disorders is rapidly increasing during aging, suggesting that alterations in metabolic control are tightly coupled to the age-associated metabolic disorders. Thus, it is not surprising that the perturbation of hypothalamic circuit activity triggered by chronic inflammation during aging has been linked to metabolic disorders. In this context, microglia activation is a crucial event in the cascade of neuronal degeneration during aging [20]. It is also well established that microglia activation triggers hypothalamic inflammation in response to metabolic enrichment and thereby leads to metabolic abnormality [21]. In this study, we successfully validated that the aged hypothalamus retains the microgliosis accompanied by the inflammatory responses. 

Many studies have focused on determining the underlying cellular mechanism of hypothalamic inflammation with great attention to the relationship between FA levels and altered metabolic control [13,18]. A majority of the studies have reported elevation in the sFA levels in the circulating system during the late period of obesity [22,23]. These observations suggested that metabolic abnormalities involved in over-nutrition are closely associated with increased levels of sFAs, active pathological substances, which elicit cellular stresses such as inflammation [24,25]. In addition, it is well known that sFAs trigger inflammatory responses in the hypothalamus as well as in peripheral organs involved in metabolic control [12,13]. Although previous literature has shown that fatty acid compositions were altered in the hippocampus of an aged rat retaining cognitive deficit [26], it has not yet been identified the profiling of fatty acid compositions in the aged hypothalamus. Intriguingly, we have currently identified that multiple sFAs were elevated in the hypothalami of aged mice, suggesting that an increase in sFAs could be a critical biochemical event that elicits disruption of normal hypothalamic functions during the aging process. We recently reported increased levels of linoleic acid, an uFA in the hypothalamus, but not in the circulating system, during short-term exposure to a HFD [14], suggesting hypothalamic tissue autonomous alteration as a mechanism to reverse the inflammatory response in association with the early over-nutrition period. Furthermore, it has been shown that brain-derived lipid content is sex-dependent [27]. In line with these findings, we demonstrated that aged mice displayed elevation in the levels of multiple sFAs in the hypothalamus, even though circulating FAs were not significantly altered. Based on the literature and our own findings, we further hypothesized that the energy utilization in hypothalamic cells during aging might be distinguishable from the other organs and blood stream. Intriguingly, we showed that the aged hypothalamus expressed higher levels of FA transporter and genes involved in FA utilization such as FA oxidation and ketogenesis. In contrast, the expression of genes involved in glucose utilization, including glycolysis and lactate synthesis, was significantly reduced in the hypothalami of aged mice. These cellular phenomena suggest that a declined glucose metabolism during aging leads to a shift from glucose to FAs as a source of energy for normal brain function. Our findings are in accordance with the literature, where it has been shown that the brain tissues during aging displayed enhanced fatty acid utilization [28]. These cellular alterations could explain the increased hypothalamic FA levels during aging. Notably, the FA profiling revealed increased levels of multiple sFAs and reduced levels of oleic acid, an uFA in the hypothalami of aged mice. Since uFA is beneficial for the anti-inflammatory response, our result might be part of a series of cellular responses coupled to the hypothalamic inflammatory responses during aging. In line with this notion, a previous study has also shown that oleic acid leads to anti-inflammatory effects in the hypothalamus and thereby improves dysfunction of the hypothalamus [29].

To conclude, our study demonstrated that the composition of FAs in aging hypothalamus is altered without changes in the levels of circulating FAs. Notably, the alterations of hypothalamic FA composition might be coupled to the development of hypothalamic inflammation linked to microglia activation. Thus, alterations in nutrient utilization and the composition of hypothalamic fatty acids might be associated with aggravation of metabolic control during the aging process. 

## 4. Materials and Methods 

### 4.1. Animals

The mice used in this study were male C57BL/6J, four-month-old (young) and twenty-four-month-old (aged), and with body weights (BWs) of 25–27 g and 35–40 g, respectively. The mice were maintained in a temperature- (23–25 °C) and humidity-controlled chamber with a 12 h light/dark cycle (light exposure during 7 am to 7 pm). The mice had access to standard chow (DBL, Eumseong, North Chungcheong Province, South Korea) and water ad libitum. Changes in BW were monitored for a month. At the end of that period, the hypothalamic tissues were quickly excised from the mice and stored at −80°C for further analysis of gene expression and long chain fatty acid profiling. All animal care and experimental procedures were approved by the Institutional Animal Care and Use Committee (IACUC) at the Incheon National University (permission number: INU-2018-03).

### 4.2. Analysis and Extraction of Long Chain Fatty Acids in Hypothalamus and Serum

The extraction and analysis of long chain fatty acids (LCFAs) from hypothalamus and serum were performed as previously described with some modifications [14]. Each hypothalamic sample was mixed with 0.7 mL of chloroform:methanol (2:1, *v*/*v*) solution, 0.1 mL pentadecanoic acid in chloroform (1 mg/mL, internal standard), and approximately 300–350 mg of glass beads (acid-washed, 425–600 μm, G8772, Sigma-Aldrich, St. Louis, MO, USA) in a tube and homogenized using a bead beater (Mini Beadbeater-96, BioSpec Products, Bartlesville, OK, USA) for 10 s. After homogenization, the samples were vortexed for 20 s, sonicated for 10 min, mixed with 0.58% sodium chloride (0.7 mL), and centrifuged at 15,000× *g* at 4 °C for 5 min. The underlayer liquids (approximately 0.45 mL) were transferred in new tubes and concentrated using a centrifugal concentrator (VS-802F, Visionbionex, Gyeonggi, Korea). In case of extraction from serum, each sample of 0.1 mL was mixed with 2.5 mL of chloroform:methanol (2:1, *v*/*v*) solution and 0.1 mL of pentadecanoic acid (1 mg/mL, IS) and sonicated for 10 min. The sonicated samples were mixed with 2.5 mL of 0.58% sodium chloride, vortexed for a few seconds, and centrifuged at 15,000× *g* for 5 min at 4 °C. The lower layer (approximately 1.8 mL) was transferred to a clean tube and dried using the centrifugal concentrator (VS-802F, Visionbionex, Gyeonggi, Korea). 

Each dried and concentrated sample obtained from the hypothalamus or serum was blended with 0.1 mL toluene, 0.02 mL of 5 M sodium hydroxide, and 0.18 mL methanol. The sample mixture was reacted at 85 °C for 5 min at 300 rpm. To catalyze the reaction, 0.3 mL of boron trifluoride was added to the tube and the above procedure was repeated. After cooling for 3 min, 0.4 mL distilled water and 0.8 mL pentane were added to the mixture, which was centrifuged for 15 min at 350× *g* and 4 °C. The supernatant was transferred to a 2 mL tube and concentrated again. The concentrated sample was dissolved in 0.1 mL hexane. The samples were filtered through a 0.5 μm syringe filter and analyzed using gas chromatography with flame ionization detection. The methylated fatty acids (1 μL) were separated using an Agilent 7890 A gas chromatograph and 7890 GC detector equipped with a DB-WAX column (30 m × 0.25 mm, 0.25 μm film thickness, Agilent Technologies, Santa Clara, CA, USA). The carrier gas was nitrogen and the flow rate was 1 mL/min. Front inlet and detector temperatures were maintained at 250 °C. The initial column temperature was 130 °C for 3 min and raised to 230 °C at a rate of 20 °C/min. The final temperature was increased from 230 to 250 °C at a rate of 3°C/min and maintained for 5 min. LCFAs were identified by comparing with fatty acid methyl ester standards. 

### 4.3. Quantitative Real-Time PCR

Total RNA was isolated from the hypothalami of young and aged mice and reverse-transcribed to obtain cDNA using a Maxime RT PreMix kit (Intron Biotechnology, Seoul, Korea). Real-time PCR amplification of the cDNA was analyzed using SYBR Green Real-time PCR Master Mix (Toyobo Co. Ltd., Osaka, Japan) and a Bio-Rad CFX 96 Real-Time Detection System (Bio-Rad Laboratories, Hercules, CA, USA). The results were analyzed using the CFX Manager software (CFX Manager 3.1, Bio-Rad Laboratories, Hercules, CA, USA) and normalized to the levels of the housekeeping genes *β-actin* and *L19*. The primers used were as follows: 

For *β-actin*: 

Forward: 5′-TAA AAC GCA GCT CAG TAA CAG TCC G-3′ 

Reverse: 5′-TGG AAT CCT GTG GCA TCC ATG AAA C-3′ 

For *L-19*:

Forward: 5′-GGT GAC CTG GAT GAG AAG GA-3′ 

Reverse: 5′-TTC AGC TTG TGG ATG TGC TC-3′ 

For *IL-1β*: 

Forward: 5′-AGG GCT TCC AAA CCT TTG AC-3′ 

Reverse: 5′-ATA CTG CCT GCC TGA AGC TCT TGT-3′ 

For *Iba-1*: 

Forward: 5′-AGC TTT TGG ACT GCT GAA GG-3′ 

Reverse: 5′-TTT GGA CGG CAG ATC CTC ATC-3′ 

For *TNF-α*: 

Forward: 5′-TGG GAC AGT GAC CTG GAC TGT-3′ 

Reverse: 5′-TTC GGA AAG CCC ATT TGA GT-3′ 

For *CD11b*:

Forward: 5′-CCA CTC ATT GTG GGC AGC TC-3′ 

Reverse: 5′-GGG CAG CTT CAT TCA TGT C-3′ 

For *COX-2*: 

Forward: 5′-TGC TGT ACA AGC AGT GGC AA-3′ 

Reverse: 5′-AGG GCT TTC AAT TCT GCA GCC A-3′ 

For *mPGES1*: 

Forward: 5′-CTG CTG GTC ATC AAG ATG TAC G-3′ 

Reverse: 5′-TGC CAG ATT TTC TCC ATG TCG-3′

For *Glut1*:

Forward: 5′-ATG GGC AAT GCA GAC TTG TG-3′ 

Reverse: 5′-ACG ATT GAT GAG CAG GAA GC-3′ 

For *Glut3*:

Forward: 5′-TCT GTT GGT GGC ATG ATT GG-3′ 

Reverse: 5′-ATG ATG GCC AGC AAG TTG AC-3′ 

For *HK1*:

Forward: 5′-AGA GGC CTA GAC CAC CTG AAT GTA A-3′ 

Reverse: 5′-ACT GTT TGG TGC ATG ATT CTG GAG-3′ 

For *LDH*:

Forward: 5′-AGC CCT GAC TGC ACC ATC ATC-3′ 

Reverse: 5′-CGG AAT CGA GCA GAA TCC AGA-3′ 

For *MCT1*:

Forward: 5′-AAT GAT CGC TGG TTG TC-3′ 

Reverse: 5′-TTG AAA GCA AGC CCA AGA CC-3′ 

For *FATP*:

Forward: 5′-GCA GCA TTG CCA ACA TGG AC-3′ 

Reverse: 5′-GTG TCC TCA TTG ACC TTG ACC AGA-3′ 

For *PPAR-α*:

Forward: 5′-TCC ATA AAT CGG CAC CAG GAA-3′ 

Reverse: 5′-ACG CTC CCG ACC CAT CTT TAG-3′ 

For *CPT1α*:

Forward: 5′-CCA GGC TAC AGT GGG ACA TT-3′ 

Reverse: 5′-GAA CTT GCC CAT GTC CTT GT-3′ 

For *HMGCS1*:

Forward: 5′-TTT GAT GCA GCT GTT TGA GG-3′ 

Reverse: 5′-CCA CCT GTA GGT CTG GCA TT-3′ 

For *HMGCL*:

Forward: 5′-CCA GCT TTG TTT CTC CCA AG-3′ 

Reverse: 5′-TCA GAC ACA GCA CCG AAG AC-3′ 

### 4.4. Immunohistochemistry (IHC)

Mice were deeply anesthetized with tribromoethanol and perfused transcardially with 0.9% saline (*w*/*v*), followed by a fresh fixative of 4% paraformaldehyde in phosphate buffer (PB, 0.1 M, pH 7.4). Brains were harvested and post-fixed overnight at 4 °C with 4% paraformaldehyde in PB before preparing the coronal sections (50 μm thickness) using a vibratome (5100 mz Campden Instruments, Leicestershire, UK). After several washes with PB, the sections were preincubated with 0.3% Triton X-100 (Sigma-Aldrich, St. Louis, MO, USA) for 30 min to permeabilize the tissues and cells at room temperature. After further washing with PB, the sections were incubated with primary antibodies against rabbit Iba-1 (1:1000 dilution, Wako, Osaka, Japan) and rabbit COX-2 (1:1000 dilution, Abcam, Cambridge, UK) overnight at room temperature. On the next day, the sections were washed thoroughly and incubated with biotinylated anti-rabbit secondary antibodies for 2 h at room temperature. Afterwards, the sections were rinsed with PB and incubated with avidin–biotin peroxidase solution (ABC Elite, Vector Laboratories, Burlingame, CA, USA) for 2 h at room temperature. Color reaction was developed using 3,3′-diaminobenzidine substrate (DAB, Vector Laboratories, Burlingame, CA, USA) in 0.01% H_2_O_2_ for 1 min. The sections were then mounted onto glass slides and covered using coverslips with a drop of mounting medium (Dako North America Inc, Carpinteria, CA, USA). The coverslips were sealed with nail polish to prevent desiccation and movement of the samples under the microscope.

### 4.5. IHC Image Capture and Analyses

The images were recorded using fluorescence microscopy (Axioplan2 Imaging, Carl Zeiss Microimaging Inc., Thornwood, NY, USA). The sections containing hypothalamic nuclei (stereotaxic coordinates: between −1.46 and −1.82 mm from the bregma) were matched with the mouse brain atlas book (Paxinos and Franklin, 2001, the Mouse Brain in Stereotaxic Coordinates-second edition, San Diego, CA, USA, Academic Press) and subjected to IHC analyses. Both sides of the bilateral brain region were analyzed (two brain sections per mouse). The number of immunostained cells, soma size, and the intensity of immunoreactive signals of Iba-1 in the microglia were measured manually using ImageJ v. 1.47 software (National Institutes of Health, Bethesda, MD, USA; http://rsbweb.nih.gov/ij/) by an unbiased observer. All morphometric analyses were performed without prior knowledge about the experimental group from which the sections were obtained (blind).

### 4.6. Statistical Analyses

Statistical analysis was performed using Prism 6.0 software (GraphPad Software, San Diego, CA, USA). An unpaired *t*-test was performed to analyze the significance (*p* < 0.05) between the aging and control groups.

## Figures and Tables

**Table 1:** Composition of fatty acids in hypothalamus and serum of young and aged mice.

Fatty Acid (µg/g)	Hypothalamus		Serum	
	4 Months (Young)	24 Months (Old)	4 Months (Young)	24 Months (Old)
C14:0 (myristic acid)	42.38 ± 3.11 ^1^	55.51 ± 3.53 *	4.02 ± 0.31	4.60 ± 0.55
C16:0 (palmitic acid, mg/g)	6.63 ± 0.16	7.21 ± 0.11 *	0.47 ± 0.05	0.44 ± 0.03
C16:1n7 (palmitoleic acid)	213.03 ± 6.78	204.30 ± 4.35	45.43 ± 8.62	45.06 ± 5.77
C18:0 (stearic acid, mg/g)	5.61 ± 0.09	5.72 ± 0.04	0.19 ± 0.01	0.19 ± 0.01
C18:1n9 (oleic acid, mg/g)	6.15 ± 0.07	5.51 ± 0.03 ***	0.22 ± 0.04	0.24 ± 0.02
C18:2n6 (linoleic acid)	200.00± 14.67	238.33 ± 5.55 *	584.20 ± 71.75	515.85 ± 47.49
C18:3n3 (α-Linolenic acid)	ND ^2^	ND	80.21 ± 5.10	74.94 ± 5.67
C18:3n6 (γ-Linolenic acid)	143.13 ± 13.58	202.73 ± 11.35 *	9.76 ± 1.00	9.69 ± 0.85
C20:0 (arachidic acid)	78.10 ± 5.44	122.05 ± 6.68 **	ND	ND
C20:3n6 (dihomo-γ-linolenic acid)	105.32 ± 5.74	114.18 ± 3.25	22.97 ± 3.20	22.36 ± 2.60
C20:4n6 (arachidonic acid, mg/g)	3.68 ± 0.07	3.78 ± 0.33	0.15 ± 0.01	0.16 ± 0.01
C20:5n3 (eicosapentaenoic acid)	182.74 ± 11.32	218.70 ± 11.53	32.89 ± 0.30	26.99 ± 1.31
C24:0 (lignoceric acid, mg/g)	4.25 ± 0.10	4.43 ± 0.06	0.08 ± 0.009	0.07 ± 0.006

^1^ Mean ± standard deviation. ^2^ Not detected. * *p* < 0.05; ** *p* < 0.01; *** *p* < 0.001; compared to young group.
